# Optimization of Charpy Impact Strength of Tough PLA Samples Produced by 3D Printing Using the Taguchi Method

**DOI:** 10.3390/polym16040459

**Published:** 2024-02-07

**Authors:** Oğuz Tunçel

**Affiliations:** Department of Mechanical Engineering, Faculty of Engineering, Siirt University, Siirt 56000, Türkiye; oguz.tuncel@siirt.edu.tr; Tel.: +90-484-212-11-11

**Keywords:** Charpy impact strength, FDM, 3D printing, tough PLA, Taguchi, ANOVA

## Abstract

This research employs the Taguchi method and analysis of variance (ANOVA) to investigate, analyze, and optimize the impact strength of tough polylactic acid (PLA) material produced through fused deposition modeling (FDM). This study explores the effect of key printing parameters—specifically, infill density, raster angle, layer height, and print speed—on Charpy impact strength. Utilizing a Taguchi L16 orthogonal array experimental design, the parameters are varied within defined ranges. The results, analyzed through signal-to-noise (S/N) ratios and ANOVA, reveal that infill density has the most substantial impact on Charpy impact strength, followed by print speed, layer height, and raster angle. ANOVA identifies infill density and print speed as the most influential factors, contributing 38.93% and 36.51%, respectively. A regression model was formulated and this model predicted the impact strength with high accuracy (R^2^ = 98.16%). The optimized parameter set obtained through the Taguchi method, namely, a 100% infill density, 45/−45° raster angle, 0.25 mm layer height, and 75 mm/s print speed, enhances the impact strength by 1.39% compared to the experimental design, resulting in an impact strength of 38.54 kJ/m^2^. Validation experiments confirmed the effectiveness of the optimized parameters.

## 1. Introduction

In today’s world, many manufacturing sectors increasingly require cost-effective, high-quality, customized components with the necessary mechanical features [1,2]. Additive manufacturing (AM) or 3D printing represents a crucial advancement in producing components featuring intricate geometries and desired characteristics [3,4]. Unlike conventional machining technologies, AM technologies enable the manufacturing of the physical part from three-dimensional computer-aided data, layer by layer, using as much material as needed [5,6]. Due to the cost-effectiveness of assembly and materials, the utilization of 3D-printed components with commercially available 3D printers employing various manufacturing technologies and materials, such as stereolithography (SLA), digital light processing (DLP), binder jetting (BJ), and fused deposition modeling (FDM), is on the rise [7,8]. 

The FDM method is currently prevalent due to the development of affordable devices, the fact that it does not require health-harmful solvents and adhesives during sample production, the inexpensive cost of printing materials, and the small size of the devices able to fit on a tabletop [9]. The FDM process involves using different thermoplastic materials to produce objects with complex shapes [10]. Polylactic acid, known as PLA, is a specialized thermoplastic material that can be sourced from renewable raw materials like cornstarch or sugarcane, rendering it sustainable. Its classification as a bioplastic stems from its origin in renewable sources rather than petroleum. PLA finds extensive use in several medical instruments, including implants, dentistry, drug carriers, and orthopedic interventions, and in food wrapping boxes. The favor of PLA filaments in 3D printing applications is attributed to the ease of printing with these filaments [11,12,13]. Nevertheless, PLA filaments employed in FDM technology have shortcomings that adversely impact brittleness, abrasion resistance, fracture toughness, and impact resistance. Tough PLA has been developed to overcome these limitations and is attracting much attention. In particular, this material offers unique properties such as improved toughness and impact resistance. With an energy absorption capacity three times higher than standard PLA filaments, tough PLA offers excellent flexibility, high abrasion, and impact resistance, making it highly suitable for various engineering applications [14,15,16,17]. Impact resistance studies are frequently conducted to determine the fracture toughness of composite PLA parts. However, there is a lack of studies on the parameters affecting the impact resistance of tough PLA materials.

Through a literature review conducted before the experimental studies, recent studies on the mechanical properties and optimization of FDM parameters of 3D-printed parts are summarized below. Beylergil et al. [18] used Taguchi optimization to enhance Charpy impact strength in 3D-printed CF/PA composites. Optimal parameters resulted in a 150% increase in impact strength (10.54 kJ/m^2^). Ansari et al. [19] obtained noteworthy results in their study on the impact of 3D printing on CF-reinforced PLA fabrication. Optimized parameters achieved maximum impact strength (113.84 J/m) and hardness (79.6 Shore D). Atakok et al. [20] utilized the Taguchi methodology to evaluate the impact of FDM parameters on PLA and recycled PLA (Re-PLA) properties. The results highlighted layer thickness as the most effective factor, showcasing improved mechanical features (16.96 kJ/m^2^ impact strength) with optimal values. SEM analysis confirmed the feasibility and environmental benefits of 3D printing with Re-PLA filaments. Erkendirci’s study [21] on glass-reinforced composites revealed that increasing thickness and volume fraction resulted in higher impact energies and toughness. Damage mechanisms included matrix, fiber fractures, and fiber pull-out in all laminates. Kam et al. [22] determined in their studies that the layer thickness significantly influences the impact strength of PA12 specimens that are 3D-printed by FDM. The optimal impact strength (25.3 kJ/m^2^) was found with a layer height of 0.25 mm, a 50% infill rate, a rectilinear filling structure, and a print temperature of 250 °C. Lee and Wu [23] obtained the following results in their study: the 3D-printed CFR-PLA composites exhibited a rougher surface topology compared to pure PLA, with the table temperature found to be the most influential factor for tensile strength and both bed temperature and print orientation affecting impact strengths. Parts printed at a 45° orientation demonstrated superior mechanical strength to those printed at a 90° orientation. Lambiase et al. [24] found a significant effect of raster angle on the fracture toughness of additively manufactured double cantilever beam specimens. Their results, illustrated in plots of energy release rates for crack initiation and propagation, indicated improved toughness with monodirectional deposition when filaments are oriented perpendicular to crack propagation. Notably, superior fracture toughness levels were achieved with alternating deposition strategies, particularly at ±45° raster angles. Sharif et al.’s PLA study [25] on FDM-based additive manufacturing found that a higher infill density (40–80%) increased impact strength, but thicker layers and higher printing speeds decreased it. The optimal conditions were 80% infill, 0.1 mm thickness, and 100 mm/s speed (22.12 kJ/m^2^). Layer thickness and infill rate significantly contributed to a higher impact strength. In their study, Zisopol et al. [26] achieved significant results, demonstrating that infill percentage plays a crucial role in the impact resistance of annealed PLA 3D-printed pieces, particularly with 100% infill. High impact strengths of up to 9.69 kJ/m^2^ were obtained particularly in annealed specimens. Tanveer et al. [27] reported a range of tensile strength values for PLA specimens with varying infill density, from the highest at 46.3 N/mm^2^ to the lowest at 29.9 N/mm^2^. The impact strength results showed the highest Charpy strength at 4.72 kJ/m^2^ and the lowest Izod strength at 1.7 kJ/m^2^. These findings indicated a positive correlation between infill density and tensile strength, while a reduction in impact strength was observed with varying infill density. Nagendra and Prasad [28] achieved notable improvements in FDM-printed nylon parts by optimizing process parameters. The optimal settings, including a layer height of 0.4 mm, an extruder temperature of 300 °C, 90% density, a 90-degree raster angle, and a rectilinear filling model, enhanced mechanical properties. The test specimen at these settings exhibited a 7.2% increase in tensile strength (51.455 MPa), 22.7% in flexural strength (98.164 MPa), 27.4% in impact strength (0.637 MJ/m^2^), and 7.5% in compressive strength (19.42 MPa) compared to parts printed with standard settings using pure nylon. Solouki et al. [29] achieved optimal FDM parameters for PLA parts, discovering that a layer thickness of 0.3 mm, two top and bottom layers, 60% density, and a print rate of 45.28 mm/s provided the best overall performance. Layer height emerged as the most influential parameter, impacting part strength, production time, weight, and dimensional accuracy.

Various researchers have investigated the effects of 3D printing parameters on the behavior of PLA and other composite materials, as described in the paragraph above. The study of printing parameters, mainly mechanical properties, has been particularly intense. Tough PLA is a material developed to address the weaknesses of traditional PLA. It is less brittle and more resistant to abrasion and impact. However, the evaluation of the effects of 3D printing parameters on the impact strength of high-toughness PLA materials has yet to be extensively investigated. Therefore, there is a pressing need for additional investigations to unravel the nuances of how various 3D printing process parameters affect the impact resistance of tough PLA materials. This study examines the impact of 3D printing parameters on the Charpy impact strength of tough PLA materials. The goal is to optimize parameters that have yet to be adequately studied on this particular material.

The experimental design and statistical analysis were performed using the Taguchi L16 orthogonal array to plan the experiments. Four specific parameters related to 3D printing were investigated: infill density, raster angle, layer height, and print speed. The experimental results were interpreted using the signal-to-noise ratio (S/N), analysis of variance (ANOVA), interactions between the print parameters, a contour plot, and a mathematical model for Charpy impact strength derived at a 95% confidence level by regression analysis. At the end of the study, validation experiments were conducted to determine the optimal combinations, and the effect of the optimized parameters on impact strength was indicated. Fracture surfaces were also examined using scanning electron microscopy (SEM). In conclusion, the scientific value of this work is that it systematically and comprehensively addresses the influence of 3D printing parameters on the impact strength of tough PLA materials and presents optimized parameter combinations for industrial applications. This is essential in producing cost-effective, high-quality, and customizable components in the manufacturing sector.

## 2. Materials and Methods

**Figure 1:**
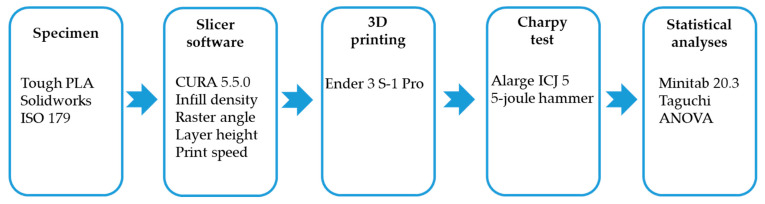
Experimental procedure.

This study utilized tough PLA filament, developed as an alternative to difficult-to-print ABS filaments for professional users, increasing the impact strength by about three times compared to standard PLA filaments [30,31]. Tough PLA overcomes the shrinkage and warping problems of ABS material with the ease of printing. Tough PLA has better-sanding properties than standard PLA filaments. It is preferred in applications where impact resistance is required. A comparison of the physical, thermal, and mechanical properties of PLA and tough PLA filaments produced by Porima (Yalova, Türkiye) is shown in Table 1. In addition, the experimental procedure used in this study is visualized in Figure 1, from the design of the sample to the statistical analysis.

**Figure 2:**
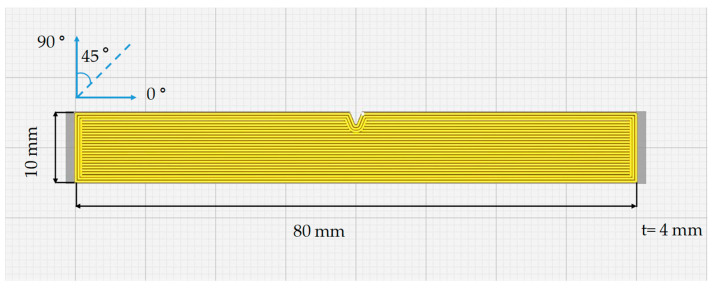
A schematic representation of the first layer print and raster angle of the sample prepared according to ISO 179. (t = thickness).

The 3D model of the notched Charpy impact sample was modeled according to the ISO 179 standard, utilizing Solidworks 2020 software. According to this standard, the specimen was 80 mm in length, 10 mm in width, and 4 mm thick [32,33]. A slicing program was needed to print the designed impact specimen on a 3D printer and to set the printing parameters precisely. The 3D model was sliced using Ultimaker CURA 5.5.0 software. Figure 2 shows a screencap of the slicer window, showing a notched Charpy impact test specimen with a line-type fill pattern and a scan angle orientation of 0°.

**Figure 3:**
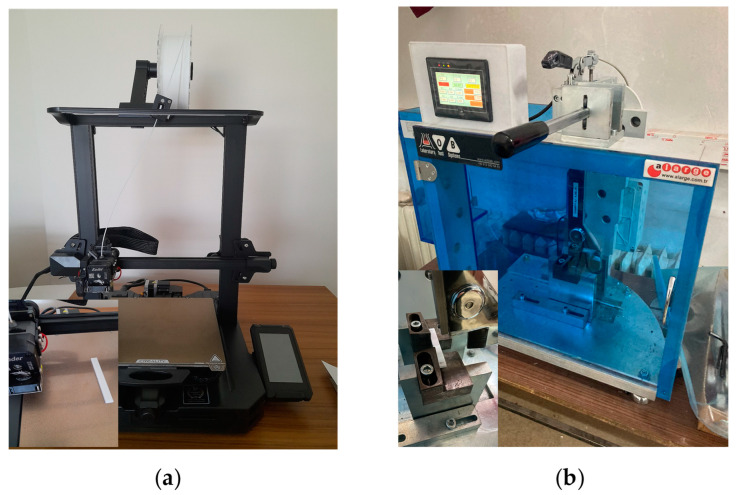
(**a**) Creality Ender-3 S1 Pro printer for sample production; (**b**) Alarge IJC 5 model impact tester.

The test specimens were fabricated using the Creality Ender-3 S1 Pro (Shenzhen, China) FDM 3D printing system. The 3D printer used to produce the specimens is shown in Figure 3a. The fixed printing parameters in Table 2 were used in the 3D printing processes. The FDM system specifications are also presented in this table.

Impact testing was performed to measure the response of a material to impact loads that can lead to sudden deformation or complete rupture and to determine the energy absorbed. The fracture energy is usually determined by releasing a heavy hammer at the end of a pendulum to collide with the specimen supported in the fixture. In the current study, Charpy impact tests were executed in a laboratory environment at room temperature and 50% relative humidity using an Alarge ICJ 5 impact tester (Alarge Company, Istanbul, Türkiye) with a 5-Joule hammer, with five repetitions from each group. Figure 3b demonstrates the Charpy impact machine. During the impact test, the pendulum speed reached 2.9 m/s, and the sample was subjected to a rapid and intense impact by the hammer pendulum. The principle of determining the impact energy is based on using the potential energy difference between the starting and ending positions of the hammer. Based on this principle, the impact energy is calculated using Equation (1) below: E is the absorbed impact energy (Joule), b is the width of the specimen up to the notch base (mm), and h is the specimen thickness (mm) [34,35].

(1) $$Charpy\;impact\;strength\;=\frac{E}{b.h}\times \;10^{3}(\mathrm{kJ}/m^{2})$$

In this study, the Taguchi experimental method was designed and applied to evaluate the effect of print parameters such as infill density (%), raster angle (°), layer height (mm), and print speed (mm/s) on the impact strength of FDM-manufactured tough PLA parts. The Taguchi approach to experimental design utilizes orthogonal arrays to arrange performance parameters and their corresponding levels for variation systematically. The Taguchi method aims to determine product quality and procedures with a minimum number of experiments to save cost and time. In contrast to a comprehensive full factorial design that explores all possible combinations, the Taguchi technique assesses pairs of possibilities. Table 3 illustrates this approach, where process parameters were defined with four parameters and four levels. According to Table 3, 256 (4^4^) test groups and 1280 test specimens in total would have been used if the experiments were performed with the full factorial method. Instead of this time-consuming and expensive method, the Taguchi L16 orthogonal array was applied in this study using Minitab 20 software. With the Taguchi method, only 80 (16 × 5) samples were manufactured to analyze the effect of the parameters.

The Taguchi method transforms results from orthogonal experimental design into a signal-to-noise (S/N) ratio, expressed in decibels (dB). The signal factor expresses the actual value obtained in the system. In contrast, the noise factor relates to factors that are not considered in the experiment setup but have an impact on the result. Noise sources are all variables that cause deviations from the target value. Three aim functions are generally used in S/N ratio analysis: “nominal is better”, “larger is better”, and “lower is better”. In this study, the experiment results were analyzed using the approach that calculates the signal-to-noise ratio based on the ‘larger value is better’ criterion, aiming to obtain the maximum values for all experimental outcomes. In Equation (2), n is the number of experiments, and y_i_ is the i data obtained [36,37,38].

(2) $$\frac{S}{N_{\max}}=\;-10\log\left(\frac{1}{n}\sum_{i=1}^{n}\frac{1}{y_{i}^{2}}\right)$$

**Figure 4:**
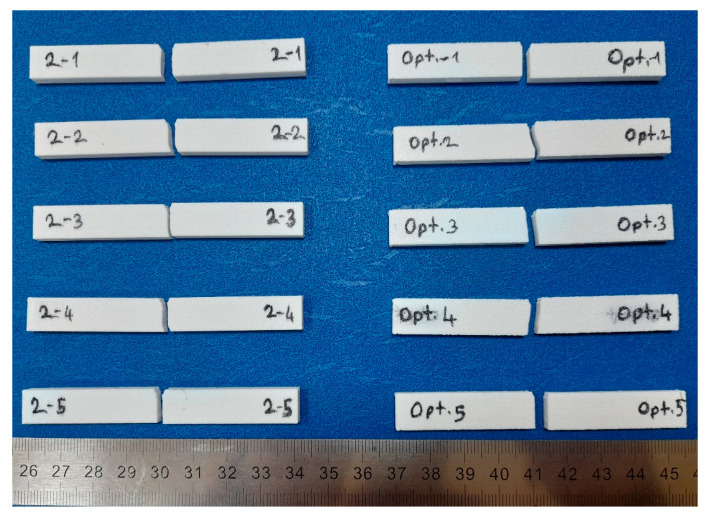
Image of fracture specimens after Charpy impact test.

Figure 4 shows the images of the fractured specimens after the Charpy impact test. The lowest and highest Charpy impact specimens were selected. Scanning electron microscope (SEM) images of the fracture surfaces of DOE-2 and optimum specimens and their explanations are presented in the results and discussion section. A ZEISS EVO 40 SEM (Carl Zeiss, Oberkochen, Germany) capable of morphological imaging up to 40.000× was used to examine the crack surfaces.

## 3. Results and Discussion

In this study, the variation in and optimization of the impact strength of tough PLA materials with printing parameters were determined by applying the Taguchi method. In the Taguchi method, three essential instruments, such as orthogonal experimental design, S/N ratio, and ANOVA, were used to analyze and evaluate the numerical results. The Taguchi method employs the signal-to-noise (S/N) ratio as a statistical metric that determines performance characteristics. It operates as a logarithmic expression of the targeted response, defined as the objective function [39,40]. This study selected the impact strength obtained from the Charpy impact test applied to the FDM-produced tough PLA specimen groups as the performance characteristic. High Charpy impact strength will ensure that the specimens are stable against an impact; thus, “larger is better” was chosen as the quality characteristic. Table 4 outlines the mean, standard deviations, and S/N ratios for the Charpy impact test outcomes across all experimental groups in the design. The tests yielded average Charpy impact strength values ranging from 34.66 kJ/m^2^ to 38.01 kJ/m^2^. The difference between the lowest and highest values of Charpy impact strength is 9.7%. The standard deviations for all experimental groups are in the acceptable range from 0.85% to 1.68% for Charpy impact tests. Compared to the studies on the Charpy impact strength of PLA produced by FDM mentioned in the introduction, the overall impact strength values for tough PLA are quite high.

**Figure 5:**
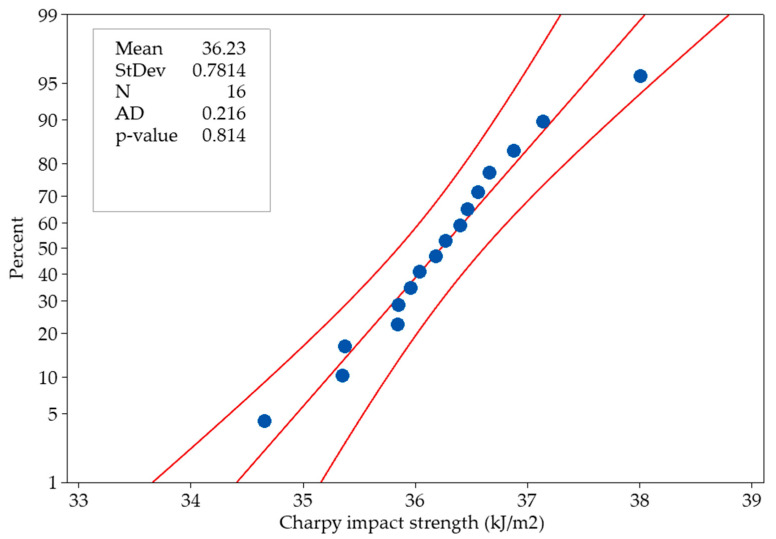
Normal probability plot of Charpy impact strength at 95% confidence interval.

Figure 5 shows the impact strength values obtained from the Charpy impact test specimens produced within the selected parameters, corresponding to the percentages of the normal distribution. The stability of the experimental design is related to the closeness of the outputs to the middle axis. In the normality test, *p* ≥ 0.05 is required for the hypothesis to be accepted [41]. In Figure 5, the *p*-value is 0.814, higher than 0.05. The data follow the normal distribution in this case, and the hypothesis is accepted.

In Taguchi optimization, the S/N response table defines and analyzes each parameter. This table contains reference results for selecting the best levels. In experimental studies, these tables determine how the data affect the desired result. The response table showing how the parameters in this study affect the Charpy impact strength is given in Table 5. When the data in Table 5 are examined, the highest values indicate optimum results according to the “larger is better” criterion selected for optimization. Also, the difference between the maximum and minimum values (delta) in this table can be used to determine the effect ratio of the parameters. The order of effect of the parameters is obtained by ranking the delta value from largest to smallest. The printing speed significantly affects Charpy impact strength (ΔS/N = 0.30 dB), closely followed by filler density (ΔS/N = 0.29 dB), while layer height has a noteworthy effect (ΔS/N = 0.22 dB), and the scanning angle has the most negligible impact (ΔS/N = 0.09 dB).

**Figure 6:**
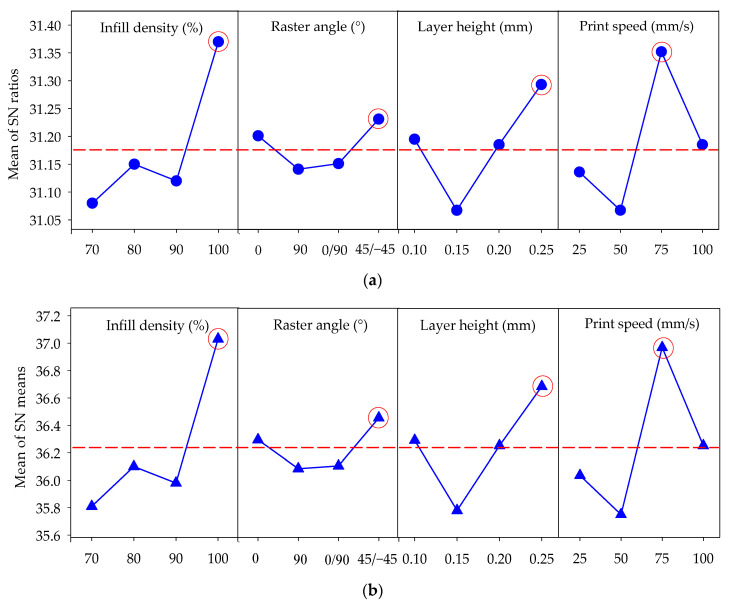
Variation in Charpy impact strength with inputs ((**a**) according to S/N ratio and (**b**) mean).

When the values in Table 5 are considered, the S/N ratios providing the optimum Charpy impact strength levels were A4, B4, C4, and D3. Meaning that 31.37 (level 4), 31.23 (level 4), 31.29 (level 4), and 31.35 (level 3) were the optimum values for infill density, raster angle, layer height, and print speed, respectively. The main effect plot for S/N ratios using the values in Table 5 is given in Figure 6a. As with the S/N ratios table, the main effect plot in Figure 6a reveals that the highest S/N values indicate optimum levels for the parameters used in FDM printing. In other words, for the highest Charpy impact strength value (kJ/m^2^), a 100% infill density, 45/−45° raster angle, 0.25 mm layer height, and 75 mm/s printing speed are the optimum printing parameter levels. Additionally, it is evident that a high infill density level, high layer height, and a printing speed of 75 mm/s increase the Charpy impact strength of tough PLA materials. The decrease in the percentage of voids and porosity and the increase in material density compared to other levels at 100% infill density highly influence impact strength [42]. A study by Kumar et al. [43] on the mechanical properties of PLA composites, similar to this study, found a decrease in impact strength above a 75 mm/s print speed and an increase in impact strength at filler densities of 70% and above.

An analysis was conducted to examine the primary impacts of the process parameters on the average response (Figure 6b). The average value at different parameter levels is called the mean response. In this context, the mean values calculated for various levels of each parameter are shown in Figure 6b. The analysis of the mean response also reveals alignment with the optimum levels identified in the S/N ratio analysis, as depicted by parameters A4, B4, C4, and D3. The average lines for these parameters are presented in Figure 6a,b. Levels above these lines indicate parameter levels that may be sufficient for high impact strength, while those below these lines may be insufficient to achieve high impact strength.

Analysis of variance (ANOVA) was applied to determine the individual effect of each printing parameter on Charpy impact strength. ANOVA, a statistical method, provides meaningful information by analyzing experimental datasets. It is valuable in determining the significance of parameter influence or interactions on a specific response [44,45]. Table 6 presents the ANOVA analysis, illustrating the scope of effects, variability, and contributions of parameters and their ratios to the experimental parameter error. ANOVA determines the variance ratio (F-value), the ratio between the regression mean square and the mean square error. This ratio reflects the factor attributable to the error term and the factor attributable to the effect of variance. A calculated high F-value indicates the significance of the parameter at the desired level. In determining this value, the F-value results are compared with each other. The ANOVA procedure was applied at a 95% confidence level. The *p*-value is used to assess the importance of a parameter. A parameter is considered statistically significant if its *p*-value is less than 0.05 [46,47]. When the ANOVA results in Table 6 were analyzed, the infill density was the most influential parameter on Charpy impact strength, with a rate of 38.93%. Similarly, print speed was also very effective, contributing 36.51%. In addition, layer height was also found to contribute 18.69%. Nonetheless, the impact of the raster angle was deemed statistically insignificant, as the *p*-value exceeded 0.05. In the parametric study by Beylergil et al. [18] on the Charpy impact strength of FDM-fabricated parts using a carbon fiber/polyamide composite, the infill density was the most influential parameter, with a high ratio of 54%. This ratio depends on the increase in the filling density of the impact specimens and the increase in plastic and carbon fibers. The current investigation yielded comparable findings.

**Figure 7:**
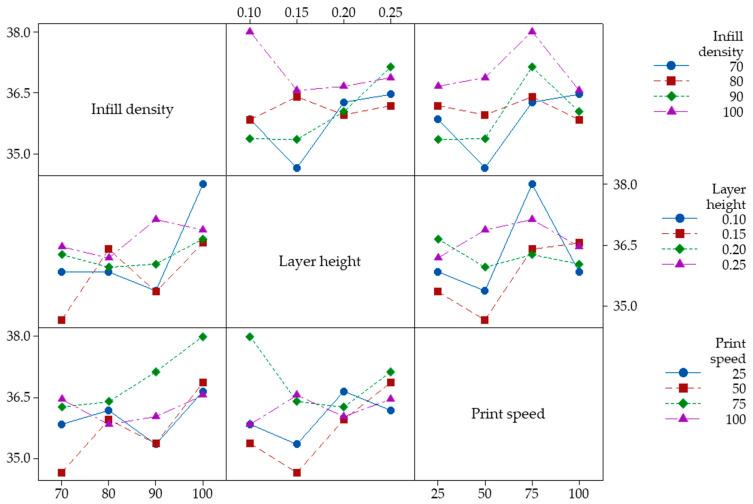
Interaction of Charpy impact strength with input parameters.

Interaction plots allow us to analyze the effect of the interaction between two variables on the Charpy impact strength values, keeping the other variables constant at their optimized levels. In interaction graphs, two graph lines are formed: parallel lines showing no interaction between the parameters and non-parallel lines showing an interaction between the parameters [48,49]. Figure 7 shows the interaction between significant parameters and represents the average effects of any two variables in all possible combinations. The raster angle found to be insignificant according to the ANOVA analysis is not accounted for here. When Figure 7 is examined in detail, it is seen that none of the trends proceed in parallel in a combined group. Because of this reason, it is concluded that there are strong interactions between the parameters analyzed within the framework of this study. When the interactions are examined, high impact strength values can generally be achieved, especially when the infill density is 100% and the print speed is 75 mm/s.

As a result of the ANOVA with a 95% confidence level, the regression model and coefficient of determination for impact strength were obtained. The R^2^ coefficient of the conclusion of the regression model for the estimated Charpy impact strength shown in Equation (3) is 0.9816. And this means that the model successfully predicted the impact strength at 98.16% (Table 6).

Predicted Charpy impact strength (kJ/m^2^) = 36.2295 − 0.417 × (A1) − 0.130 × (A2) − 0.252 × (A3) + 0.798 × (A4) + 0.064 × (B1) − 0.153 × (B2) − 0.130 × (B3) + 0.219 × (B4) + 0.041 × (C1) − 0.484 × (C2) + 0.004 × (C3) + 0.439 × (C4) − 0.216 × (D1) − 0.510 × (D2) + 0.727 × (D3) − 0.002 × (D4) (3)

**Figure 8:**
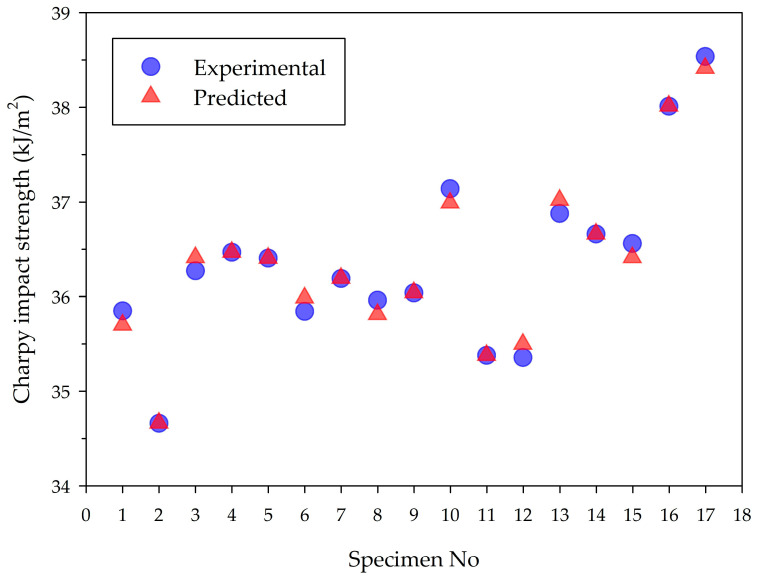
Comparison of predicted values with experimental result.

For the 16 experimental groups, the values of Charpy impact strength obtained from the experiments and predicted by the regression model are presented comparatively in Figure 8. The specimen code 17 belongs to the sample created with the optimum parameters. The average of the experimental and predicted calculated absolute percentage errors for all 16 groups in the graph is low, 0.21%. This indicates that the proposed equation successfully accurately predicts the Charpy impact strength of tough PLA materials within the specified parameters and limits.

**Figure 9:**
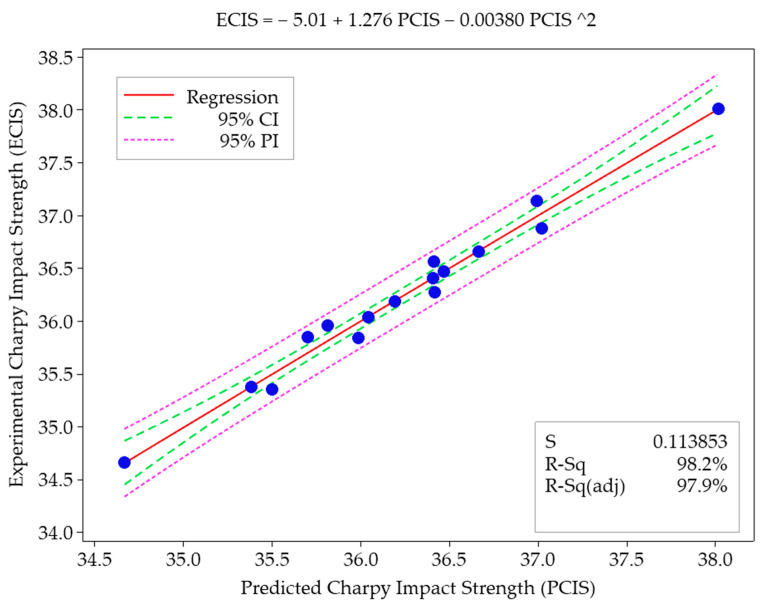
The graphical representation depicts the fitted quadratic regression line illustrating the relationship between predicted and experimental Charpy impact strength values.

Figure 9 shows the confidence intervals (CI: confidence interval) for the predicted values for all results. For this purpose, quadratic regression analysis was applied to examine the relationship between the predicted values and experimental results. The R-Sq values indicate the confidence interval of the prediction experiments. The blue dots in Figure 9 represent the experimental results, and the dashed purple lines indicate the bounds of the predicted values (PI: prediction interval). The green dashed lines represent the CI. The red straight line is the regression curve. The proximity of the experimental results to the regression curve enhances the confidence interval of the predicted values (R-Sq) [50]. When evaluating the results between experimental outcomes and predicted values for Charpy impact strength, the confidence interval (CI) limit for the predicted values is 98.2%. In comparison with the experimental results, the predicted values for Charpy impact strength fall within the confidence interval limit (95%) and are considered acceptable.

The final step of the Taguchi method optimization process involves predicting and comparing the changes in Charpy impact strength using the optimized FDM experimental input levels with the experimental results. Since the optimum values calculated by the Taguchi method were not part of the conducted experimental design, validation experiments were performed. The validation experiment was conducted on the sample created with A4B4C4D3 input levels. The results of the validation experiment are presented in Table 7. The prediction error is a very low value, 0.34%. Additionally, the impact strength of 38.54 kJ/m^2^ obtained with the optimal parameters is 1.39% higher than that of 38.01 kJ/m^2^ obtained in the experimental design of the DOE-16 sample (A4B4C1D3). The only parameter change between the two specimens was to increase the layer height from 0.1 mm to 0.25 mm. This parameter change also resulted in a limited effect of layer height on impact strength.

**Figure 10:**
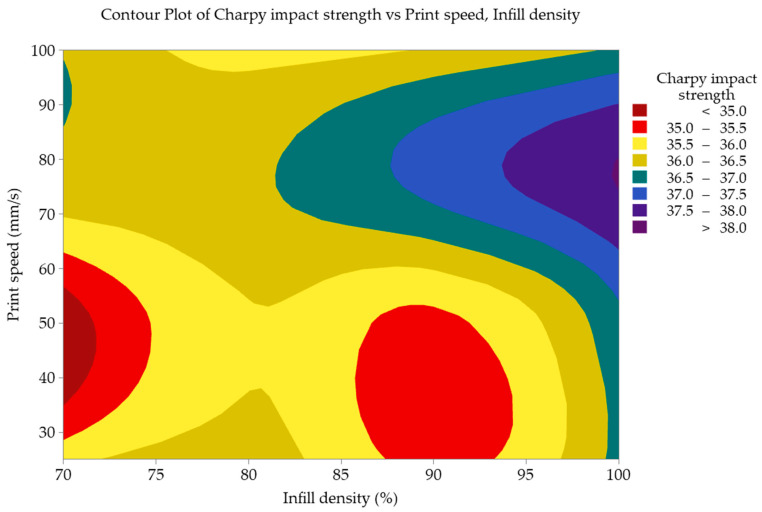
Contour plot of significant parameters (infill density and print speed).

Contour plots provide an effective visualization method to explore the correlation between a response variable and two input parameters. These plots portray the impact of input parameter values on the X and Y axes, with shaded contours indicating the values of the response variable [51]. Infill density and print speed were the most significant factors influencing the Charpy impact strength. The contour plot graph depicting the variation in impact strength values with infill density and print speed is presented in Figure 10. It is observed from the contour plot graph that impact strength values increase for high values of infill density and print speed.

**Figure 11:**
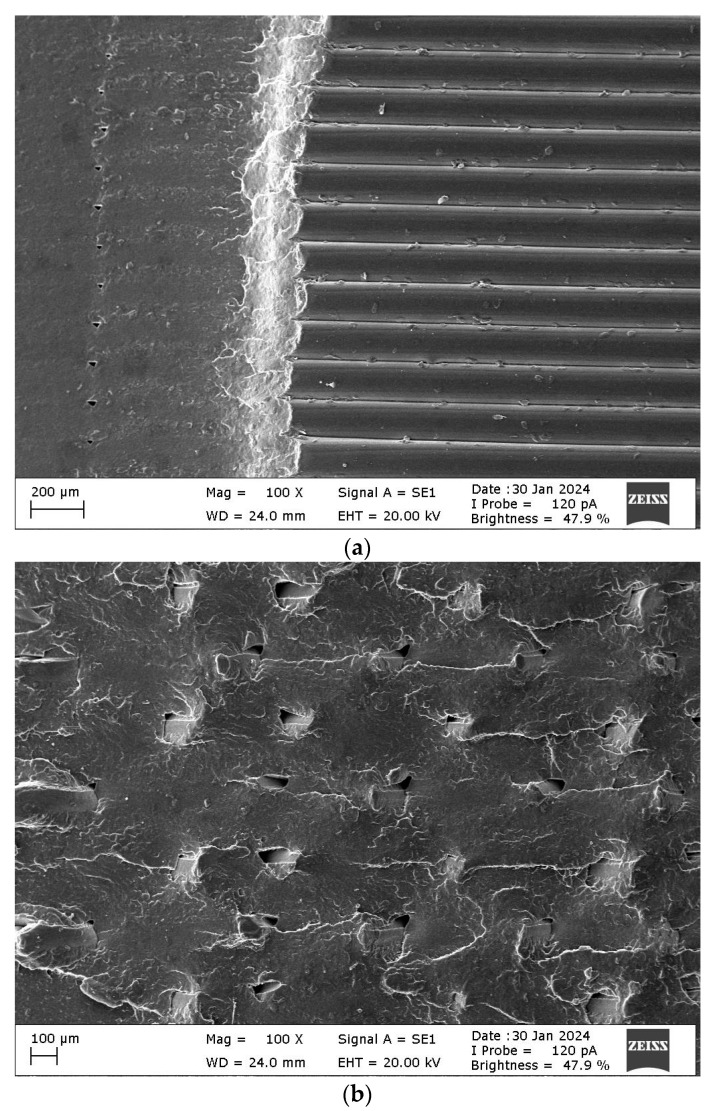
(**a**) DOE-2 and (**b**) optimum specimens’ fracture surfaces.

The fracture surfaces of the DOE-2 specimen with the lowest Charpy impact strength and the optimized specimens are shown in Figure 11. The DOE-2 sample had a 90° raster angle. Therefore, the raster direction and the direction of the hammer strike are parallel. Also, the infill density of this specimen was the lowest, at 70%. When the fracture surface of the DOE-2 specimen was examined, it was revealed that the fracture occurred abruptly in the smooth area (Figure 11a). Then, a fracture between the lines occurred because the lines were parallel to the direction of the hammer strike. The optimum specimen had a 100% infill density and a 45/−45° raster angle. Unlike DOE-2, this specimen had a rough and pitted fracture surface (Figure 11b). It is understood that the fracture is more ductile.

## 4. Conclusions

In this study, the effects of infill density, raster angle, layer height, and print speed parameters on the Charpy impact strength of tough PLA specimens produced by FDM were investigated experimentally and statistically. Statistical analyses were performed using Taguchi and ANOVA methods. The main results obtained from this study are listed below.

- Charpy impact strength results varied within a range of 9.7% (34.66 kJ/m^2^ to 38.01 kJ/m^2^).
- In Taguchi optimization, the S/N response table revealed that printing speed had the most significant effect, followed by infill density, layer height, and raster angle.
- The impact strength value of 38.54 kJ/m^2^ obtained with the optimized parameter set (A4B4C4D3) and the Taguchi method is 1.39% higher than the value of 38.01 kJ/m^2^ obtained in the experimental design. The results show that the optimum 3D printing parameters are infill density (100%), raster angle (45/−45°), layer height (0.25 mm), and print speed (75 mm/s).
- ANOVA highlighted infill density (38.93%) and print speed (36.51%) as influential factors.
- Interaction plots demonstrated strong interactions between parameters, especially emphasizing the positive impact of 100% infill density and 75 mm/s print speed on impact strength.
- The regression model successfully predicted impact strength (R^2^ = 98.16%), validated with a low prediction error (0.34%).
- The Taguchi method effectively optimized FDM parameters for enhanced tough PLA impact strength.
- Fracture surface analysis revealed that optimized specimens with higher infill density and specific raster angles exhibited more ductile fractures than lower performing specimens.

Future research could expand their scope by varying the parameters, offering a deeper exploration of how FDM printing parameters affect the mechanical properties of tough PLA. Furthermore, a comprehensive analysis would be valuable in assessing how optimized parameters enhance performance characteristics.

## Figures and Tables

**Table 1:** Technical data for PLA and tough PLA filaments.

Properties	PLA	Tough PLA
Manufacturer	Porima (Türkiye)	Porima (Türkiye)
Density (g/cm^3^)	1.23	1.22
Melt flow index (g/10min)	17.3	17.3
Heat bending temperature (°C)	55	58
Glass transition temperature (°C)	55–65	55–65
Tensile strength (MPa)	56	50
Elastic modulus (MPa)	2850	2400
Strain at break (%)	7	50
Notch impact energy (kJ/m^2^)	14.2	36

**Table 2:** FDM system properties and fixed printing parameters.

Properties	Values
Model	Creality Ender-3 S1 Pro
Printing size (mm)	220 × 220 × 270
Printing accuracy (mm)	±0.1
Filament diameter (mm)	1.75
Nozzle diameter (mm)	0.4
Print temperature (°C)	220
Table temperature (°C)	70
Top and bottom line	3
Wall line	3
Fan speed (%)	100
CAD program	Solidworks 2020
Slicer program	Ultimaker Cura 5.5.0

**Table 3:** Printing parameters and levels (Taguchi L16).

Parameters		Levels			
		1	2	3	4
A	Infill density (%)	70	80	90	100
B	Raster angle (°)	0	90	0/90	45/−45
C	Layer height (mm)	0.10	0.15	0.20	0.25
D	Print speed (mm/s)	25	50	75	100

**Table 4:** Taguchi L16 orthogonal array experimental design results of Charpy impact tests and their S/N ratios.

No	Levels				Charpy Impact Strength (kJ/m^2^)	Standard Deviation (%)	S/N (dB)
	A	B	C	D			
1	1	1	1	1	35.85 ± 0.53	1.48	31.09
2	1	2	2	2	34.66 ± 0.51	1.46	30.80
3	1	3	3	3	36.27 ± 0.44	1.21	31.19
4	1	4	4	4	36.47 ± 0.49	1.34	31.24
5	2	1	2	3	36.41 ± 0.59	1.63	31.22
6	2	2	1	4	35.84 ± 0.43	1.21	31.09
7	2	3	4	1	36.19 ± 0.34	0.93	31.17
8	2	4	3	2	35.96 ± 0.61	1.68	31.12
9	3	1	3	4	36.04 ± 0.31	0.85	31.14
10	3	2	4	3	37.14 ± 0.44	1.17	31.40
11	3	3	1	2	35.38 ± 0.50	1.41	30.98
12	3	4	2	1	35.36 ± 0.43	1.23	30.97
13	4	1	4	2	36.88 ± 0.55	1.49	31.34
14	4	2	3	1	36.66 ± 0.45	1.24	31.28
15	4	3	2	4	36.56 ± 0.47	1.28	31.26
16	4	4	1	3	38.01 ± 0.51	1.33	31.60

**Table 5:** Response table for S/N ratios of Charpy impact strength.

Level	A—Infill Density (%)	B—Raster Angle (°)	C—Layer Height (mm)	D—Print Speed (mm/s)
1	31.08	31.20	31.19	31.13
2	31.15	31.14	31.06	31.06
3	31.12	31.15	31.18	31.35
4	31.37	31.23	31.29	31.18
Delta	0.29	0.09	0.22	0.30
Rank	2	4	3	1

**Table 6:** ANOVA results for Charpy impact strength.

Source	DF	Seq SS	Adj SS	Adj MS	F-Value	*p*-Value	Contribution
Infill density (%)	3	3.5651	3.5651	1.18836	21.13	0.016	38.93%
Raster angle (°)	3	0.3690	0.3690	0.12302	2.19	0.268	4.03%
Layer height (mm)	3	1.7120	1.7120	0.57065	10.15	0.044	18.69%
Print speed (mm/s)	3	3.3433	3.3433	1.11443	19.82	0.018	36.51%
Error	3	0.1687	0.1687	0.05623			1.84%
Total	15	9.1581					100.00%
S	R-sq	R-sg (adj)	PRESS	R-sq (pred)			
0.237132	98.16%	90.79%	4.79841	47.60%			

**Table 7:** Predicted optimum value and validation of experiment result.

Optimum Level: A4B4C4D3	Predicted Value (kJ/m^2^)	Experimental Result (kJ/m^2^)
	38.41	38.54
Prediction Error (%)	0.34	

## Data Availability

Data are contained within this article.
